# Polycyclic Aromatic Hydrocarbon-Induced Signaling Events Relevant to Inflammation and Tumorigenesis in Lung Cells Are Dependent on Molecular Structure

**DOI:** 10.1371/journal.pone.0065150

**Published:** 2013-06-03

**Authors:** Ross S. Osgood, Brad L. Upham, Thomas Hill, Katherine L. Helms, Kalpana Velmurugan, Pavel Babica, Alison K. Bauer

**Affiliations:** 1 Department of Pharmaceutical Sciences, University of Colorado Anschutz Medical Center, Aurora, Colorado, United States of America; 2 Department of Environmental and Occupational Health, University of Colorado Anschutz Medical Center, Aurora, Colorado, United States of America; 3 Department of Pediatrics and Human Development, Michigan State University, East Lansing, Michigan, United States of America; University Paris Diderot-Paris 7, France

## Abstract

Polycyclic aromatic hydrocarbons (PAHs) are ubiquitous environmental and occupational toxicants, which are a major human health concern in the U.S. and abroad. Previous research has focused on the genotoxic events caused by high molecular weight PAHs, but not on non-genotoxic events elicited by low molecular weight PAHs. We used an isomeric pair of low molecular weight PAHs, namely 1-Methylanthracene (1-MeA) and 2-Methylanthracene (2-MeA), in which only 1-MeA possessed a bay-like region, and hypothesized that 1-MeA, but not 2-MeA, would affect non-genotoxic endpoints relevant to tumor promotion in murine C10 lung cells, a non-tumorigenic type II alveolar pneumocyte and progenitor cell type of lung adenocarcinoma. The non-genotoxic endpoints assessed were dysregulation of gap junction intercellular communication function and changes in the major pulmonary connexin protein, connexin 43, using fluorescent redistribution and immunoblots, activation of mitogen activated protein kinases (MAPK) using phosphospecific MAPK antibodies for immunoblots, and induction of inflammatory genes using quantitative RT-PCR. 2-MeA had no effect on any of the endpoints, but 1-MeA dysregulated gap junctional communication in a dose and time dependent manner, reduced connexin 43 protein expression, and altered membrane localization. 1-MeA also activated ERK1/2 and p38 MAP kinases. Inflammatory genes, such as cyclooxygenase 2, and chemokine ligand 2 (macrophage chemoattractant 2), were also upregulated in response to 1-MeA only. These results indicate a possible structure-activity relationship of these low molecular weight PAHs relevant to non-genotoxic endpoints of the promoting aspects of cancer. Therefore, our novel findings may improve the ability to predict outcomes for future studies with additional toxicants and mixtures, identify novel targets for biomarkers and chemotherapeutics, and have possible implications for future risk assessment for these PAHs.

## Introduction

Polycyclic aromatic hydrocarbons (PAHs) are ubiquitous environmental toxicants found in air, water, plants, soil, and sediment in many countries. Occupational exposures to PAH are due to diesel exhaust, mining activities, and oil production. While genotoxic effects of PAHs have been extensively studied, diseases such as cancer are the consequence of reversible, non-genotoxic events, ie. tumor promotion as well as irreversible mutagenic events [1]–[3]. High molecular weight (HMW) PAHs, such as benzo[a]pyrene (BaP), tend to elicit genotoxic effects while the lower molecular weight (LMW) PAHs have little to no observed carcinogenic initiation or genotoxic activity [4]–[6]. The two-four ring LMW PAHs are the most abundant PAHs in sidestream smoke or environmental tobacco smoke (ETS), reaching levels >5,500 ng/cigarette, however, little is known about these PAHs and their potential as cancer promoters. While secondhand smoke exposure has greatly decreased in the U.S., except in apartment dwellings [7], other countries, such as China, Korea, Japan, India, Russia, Poland, and Egypt are still dealing with the effects of ETS, including childhood and adult asthma, chronic obstructive pulmonary disease (COPD), and cancer, as well as other associated etiologies such as reproductive health issues [8]–[10]. Both *in vivo* and *in vitro* evidence in several cell types suggests that these non-genotoxic PAHs can modulate mechanisms involved in pulmonary diseases, such as MAP kinases (MAPK), inflammatory signaling, and influence understudied signaling events such as gap junctional intercellular communication (GJIC) [11], [12].

Alveolar type II pneumocyte is an epithelial cell type involved in many pulmonary diseases, such as asthma [13] and COPD [14], and is a progenitor cell for lung adenocarcinoma (AC) in humans and mice, which is the most common type of lung cancer in both smokers and non-smokers [15], [16]. Non-tumorigenic C10 cells used in these studies were derived from type II cells in a BALB/c mouse and have been well characterized for basal and stimulated phenotypes [17]–[20], including contact growth inhibition.

Some of the mechanisms involved in pulmonary diseases, such as idiopathic pulmonary fibrosis (IPF) and cancer, include activation of mitogenic signal transduction pathways, dyregulation of GJIC [21], and induction of inflammation pathways [1]–[3], [22]–[24], which likely interact to elicit the observed effects (eg. promotion of initiated cells during carcinogenesis). Gap junctions, composed of connexins, are intercellular channels that allow for molecular communication between neighboring cells that are often inhibited by tumor promoters [3], however very little is known about their function in other pulmonary diseases. In a study that evaluated 251 chemicals, a stronger significant correlation was observed between tumorigenicity and GJIC than with that observed with mutagenicity, suggesting that GJIC is a valid marker for promotion [24]. Tobacco smoke condensates and specific LMW PAHs in cigarettes, such as 1-Methylanthracene (1-MeA), as well as 12-O-tetradecanoylphorbol-13-acetate (TPA), a classic tumor promoter in several organs, have induced significant GJIC dysregulation in a liver cell line (WB-F44) [25]–[27]. Connexin 43 (Cx43) is the primary connexin expressed in alveolar type II and bronchiolar Clara cells [28], [29], and its expression is significantly reduced in mouse C10 cells treated with the lung tumor promoter, butylated hydroxytoluene (BHT) [28]. *Cx43^−/+^* mice also have significantly increased urethane-induced lung tumor susceptibility [30], further suggesting a role for gap junctions in pulmonary carcinogenesis and other pulmonary diseases.

MAPK pathways are also activated in response to PAHs in liver and smooth muscle cells [11], [31]. In mouse alveolar type II cell lines (C10 and E10), ERK1/2 MAPK inhibition lead to decreased cell proliferation and in mouse lung, tumor regression and restored apoptosis [19], [32]. In addition, multiple *in vivo* and *in vitro* studies have linked inflammatory pathways upstream and downstream of MAPK with lung disease mechanisms for fibrosis [33], COPD [34], and cancer (ie. tumor promotion) [3], [23], [35]–[40]. For example, Mcp-1(Ccl2), a known macrophage chemoattractant secreted in lungs by pulmonary epithelial cells, has been reported to affect MAPK activation [39].

**Figure 1:**
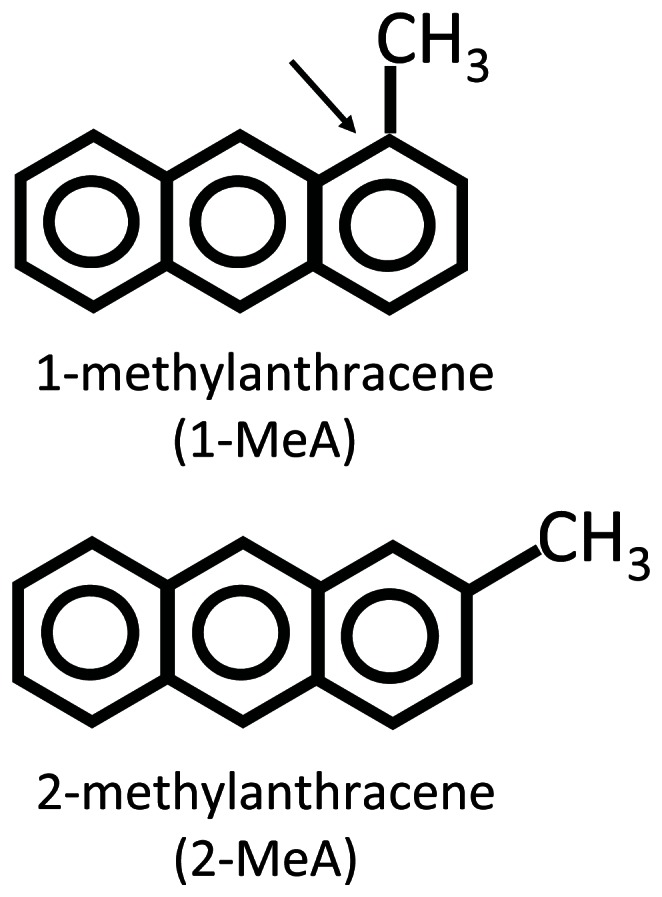
Structures of 1- and 2-methylanthracene. An arrow indicates the bay-like region of 1-MeA.

The PAH isomers herein, 1- and 2-MeA, have been shown to have disparate effects on the dysregulation of GJIC, activation of MAPK and induction of arachidonic acid release in WB-F44 rat liver cells [25]. The active GJIC inhibitor, 1-MeA, contains a bay-like structural region while the inactive isomer 2-MeA, does not (Fig. 1) [25], [41], [42]. These structural differences exist among other isomeric PAHs [43] and produced similar isomeric disparities in gap junction dysregulation as well as induction of apoptosis in a human monocyte cell line [44]. We hypothesized that the active PAH (1-MeA) would also inhibit GJIC, activate MAPKs, and induce inflammatory cytokines and chemokines, in a mouse type II cell line in contrast to its isomer (2-MeA).

## Materials and Methods

### Materials and Reagents

Reagents were purchased from Crescent Chemical (1-MeA; purity 99.5%) and Sigma Aldrich (St. Louis, MO; 2-MeA, purity 97%; acetonitrile, lucifer yellow, 12-O-tetradecanoylphorbol-13-acetate (TPA)). Stock solutions for PAHs and TPA were prepared by dissolving the compounds in acetonitrile or DMSO, respectively, which were also used as the vehicle controls. TEMED (USA) and acrylamide (China) were both purchased from BioRad (Hercules, CA). The p38 inhibitors (SB203580 and SB202190), ERK1/2 inhibitor (FR180204) and the MEK inhibitor (U0126) were purchased from Tocris Bioscience (Bristol, UK). All other reagents were purchased from Sigma Aldrich.

### Cell Line Maintenance and Treatment with PAHs

The C10 mouse cell line was obtained from Dr. Lori Dwyer Nield (University of Colorado, Aurora, CO) [18]. C10 cells are cultured in CMRL 1066 (Gibco, Invitrogen, Grand Island, NY) with 10% FBS and 1% glutamate. Cells where grown in 35 mm tissue culture dishes for the scrape load-dye transfer (SL/DT) assays and 60 mm plates for the protein and RNA extractions in a humidified atmosphere at 37°C with 5% CO_2_ and 95% air. At confluence (2–3 days), serum deprivation was initiated for 24 h prior to experimental treatments. After serum deprivation, PAHs or vehicle (CH_3_CN) were applied directly to the plates without media change from a concentrated stock solution for all experiments.

### Scrape-load/dye-transfer (SL/DT) Assay

The SL/DT assay was conducted following the method of Upham [45]. Three cuts in the monolayer of cells were made with a steel scalpel in the presence of lucifer yellow (1 mg/ml of PBS) and allowed to transfer through the cells for three minutes. The cells were then fixed with 4% formalin and the dye spread was visualized with an Eclipse Ti-S microscope. Images were collected with a DS-QiMc camera (Nikon; Melville, NY) at a magnification of 100X. The images were quantified using ImageJ software (http://imagej.nih.gov/ij/) by measuring the area of dye spread of the PAH treated plates relative to the CH_3_CN vehicle. TPA was used as a positive control for all studies; the dose was determined in previous studies (50 nM, 60 min) [46]. For the MAPK-inhibitor studies, U0126, SB203580, and SB202190 were applied separately 1 h prior to treatment with 1-MeA; doses used were previously published [19], [25], [47]. Cytotoxicity assays were performed on confluent cells after 24 h serum deprivation followed by treatment with vehicle control or PAHs for 30 min, 1.5 h, and 6 h time points. The CellTiter 96 AQueous One Solution Cell Viability assay was then performed as described by the manufacturer and measured at 490 nm (MTS assay, Promega, Madison, WI).

### Protein Extraction and Immunoblots

Cells were exposed to PAH at set intervals of 15 min to 4 h, and then extracted with 20% SDS containing protease inhibitor (Protease Inhibitor Cocktail 100X, Sigma) and phosphatase inhibitor (Halt Phosphatase Inhibitor Cocktail 100X, Thermo Scientific). The BioRad DC protein assay was used to quantify protein. 15 µg of protein were separated on a 12.5% SDS page gel and transferred to polyvinylidene fluoride (PVDF) membrane (Millipore, Billerica, MA). Primary antibodies were incubated with the membranes overnight at 4°C, similar to Rondini et al. (2010) [39] : anti-mouse Cx43 from Millipore (monoclonal, 1∶7,500 dilution) and anti-rabbit from Cell Signaling for pP38, ERK1/2 and MAPKAPK-2 (1∶500 dilution, Cat# 9215S), total P38 (1∶1,000 dilution, Cat# 9212), pERK1/2 (1∶1,000, Cat# 4370S), and total ERK1/2 (1∶1,000, Cat#4695) pMAPKAPK-2 (1∶1,000, Cat# 3007) and total MAPKAPK-2 (1∶1,000. Cat# 3042). Secondary antibody conjugated with HRP was used (Pierce Goat anti-Rabbit for pP38, total P38, pERK1/2, and total ERK1/2 all at a dilution of 1∶7500 and for Cx43 goat anti mouse IgG-HRP from Santa Cruz at 1∶1000) and a tertiary antibody for pP38 with Streptavidin-HRP from ThermoScientific at 1∶25,000. Supersignal West Dura chemiluminescent detection was used for all proteins of interest. All immunoblots were quantified by densitometry using the BioRad Quantity One Software.

### Immunostaining

Cells were grown on cover slips in a 12 well plate for 2 days and serum deprived for 24 hours before treatment. Following treatment, the cells were washed three times with PBS and fixed with warm 4% paraformaldehyde for 30 minutes. The cells were then washed again three times with PBS, permeabilized, and blocked with 5% BSA with 0.2% Triton X-100 in PBS for 90 minutes. After 90 minutes, primary antibody for Cx43 (Transduction Laboratories, monoclonal 1∶200, Cat# C13720) was applied and incubated at 4° overnight. The following day the cells were washed with PBS and secondary antibody was applied for 90 minutes (Alexa Fluor 488 goat anti-mouse, monoclonal 1∶250, Invitrogen). The plate was again washed, coated with Prolong Gold antifade reagent containing DAPI (Invitrogen), allowed to dry overnight before it was sealed, and viewed on a confocal microscope (Nikon D-Eclipse C1, 1000X).

### RNA Isolation and Quantitative Reverse Transcriptase-polymerase Chain Reaction (qRT-PCR)

RNA isolation was performed following the manufacturer’s instructions using the Nucleospin RNA II kit (Macherey-Nagel, Duren, Germany). An aliquot (1 µg) of total lung RNA was reverse transcribed as previously published [48], [49]. The cDNA was amplified in a 20 µl volume containing gene-specific primers labeled with SYBR Green master mix (Kappa Biosystems; Woburn, MA) using an Eppendorf Mastercycler ep Realplex (Eppendorf, Hauppauge, NY). *Ccl2* (*Mcp-1*) (For) 5′-GTCACCAAGCTCAAGAGAGA-3′, (Rev) 5′-GTCACTCCTACAG AAGTGCT-3; *Ptgs2 (Cox-2)*, (For) 5′-ATTGGTGGAGAGGTGTATCC-3′, (Rev) 5′-ACACTCTGTTGTGC TCCCGAA-3′; *Tnf* (For) 5′-ACGGCATGGATCTCAAAGAC-3′, *Tnf* (Rev) 5′-AGATAGCAAATCGGCTGACG-3′. The relative quantification of gene expression was calculated from the threshold cycle (C_T_) values for each sample and normalized in relation to the expression of 18S rRNA [50] using the comparative C_T_ method.

### Statistical Analysis

Data were expressed as the group mean ± standard error of the mean (SEM). Two-way ANOVA was used to evaluate the effects of treatment (vehicle, 1-MeA, 2-MeA) and concentration or time. The Student-Newman-Keuls test was used for *a posteriori* comparisons of means (*p*<0.05). All of the statistical analyses were performed using SigmaStat 3.0 software program (SPSS Science Inc., Chicago, IL).

## Results

### GJIC in Response to LMW PAHs

**Figure 2:**
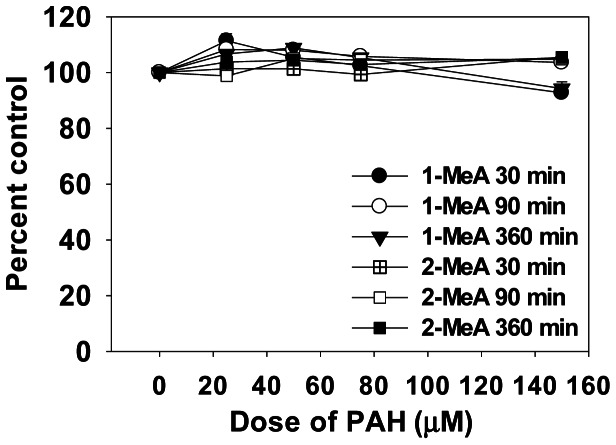
Cytotoxicity is not observed in response to 1 or 2-MeA in the C10 cells. The MTS assay observed no toxic levels of either PAH between 25–150 µM for 30 min, 1.5, or 6 h time points in the C10 cells. Mean ± SEM presented with n = 3 per study, replicated twice.

**Figure 3:**
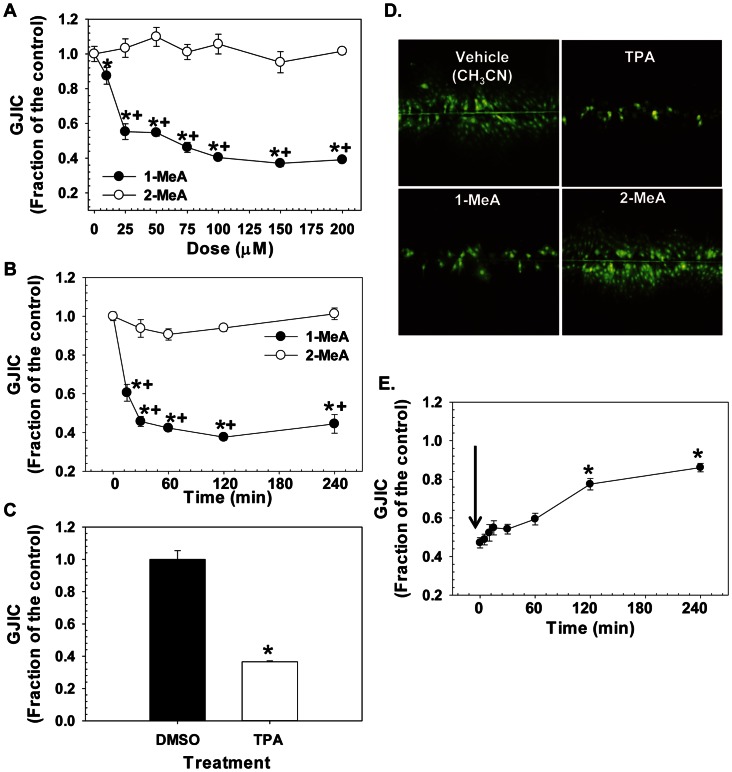
Dose response, time course, and recovery studies following treatment with 1-and 2-MeA. (A) Dose response for 1 and 2-MeA from 0–200 µM. Acetonitrile (CH_3_CN) is the vehicle control for the PAHs for all studies. (B) Time course following treatment with 1 and 2-MeA from 0–4 h. 1-MeA (75 µM) and 2-MeA (200 µM). (C) TPA positive control (50 nM, 60 min) for GJIC dysregulation in C10 cells. DMSO is the vehicle control for the TPA. Mean ± SEM presented in A–C with n = 3 per study, replicated 3 times. 1-MeA, 1-methylanthracene; 2-MeA, 2-methylanthracene. ^*^p<0.05 compared to control; ^+^p<0.05 compared to 2-MeA treated cells at the same concentration or time point. (D) Depiction of SL/DT assay after 30 min incubation with vehicle control (CH_3_CN), TPA (50 nM), 1-MeA, and 2-MeA treatment both at 75 µM demonstrating the lucifer yellow dye incorporation into the C10 cells that are communicating via gap junctions. DAPI was used for nuclear staining. Magnification at 100X. (E) Time to recovery for reversal of GJIC dysregulation in C10 cells following a 30 min treatment with 1-MeA. Black arrow indicates the removal of 1-MeA and replacement with serum-free media. Some recovery was observed approximately 1 h following removal of 1-MeA and continued through 4 h, although complete recovery was not seen. Mean ± SEM presented with n = 3 per study, replicated 3 times. ^*^p<0.05 compared to C10 cells at 0 time point which is 30 min following 1-MeA treatment.

Little cytotoxicity was observed in the C10 cells in response to either vehicle, 1- or 2-MeA treatments (Fig. 2) at doses between 25–150 µM for several time points (30 min, 1.5 and 6 h), thus all subsequent experiments on cell signaling were conducted at these non-cytotoxic doses and times. The CH_3_CN vehicle had no effect on GJIC and the positive control (TPA) demonstrated dysregulation of GJIC in C10 cells (Fig. 3C). 1-MeA was shown to inhibit GJIC in a concentration dependent manner in which the most significant change in the dysregulation of GJIC occurred between 10 and 25 µM with a maximum inhibition observed between 50–100 µM. Exposure to 2-MeA did not dysregulate GJIC, even at high concentrations (Fig. 3A). To assure robust responses for the other experiments, we chose a mid-value of 75 µM for maximum inhibition. Significant dose and treatment effects were observed for 1-MeA compared to the control or 2-MeA treatment (p<0.05). A time course experiment indicated that 1-MeA significantly and rapidly inhibited GJIC, by 15 to 30 minutes, and continued to inhibit through the 6 h time point; while 2-MeA (200 µM) did not inhibit GJIC at any of the time points (Fig. 3B). Figure 3D depicts examples of the fluorescent images of the SL-DT assay lucifer yellow dye spread following treatment of the C10 cells with 1-MeA, 2-MeA, and TPA. Since 1-MeA was shown to inhibit GJIC quickly and effectively at a concentration of 75 µM, we investigated whether this effect was reversible after removal of 1-MeA and replacement with serum free media. Significant recovery of GJIC began after 30 min, however was not completely restored 4 h later (Fig. 3E).

### MAP Kinase (MAPK) Activation

**Figure 4:**
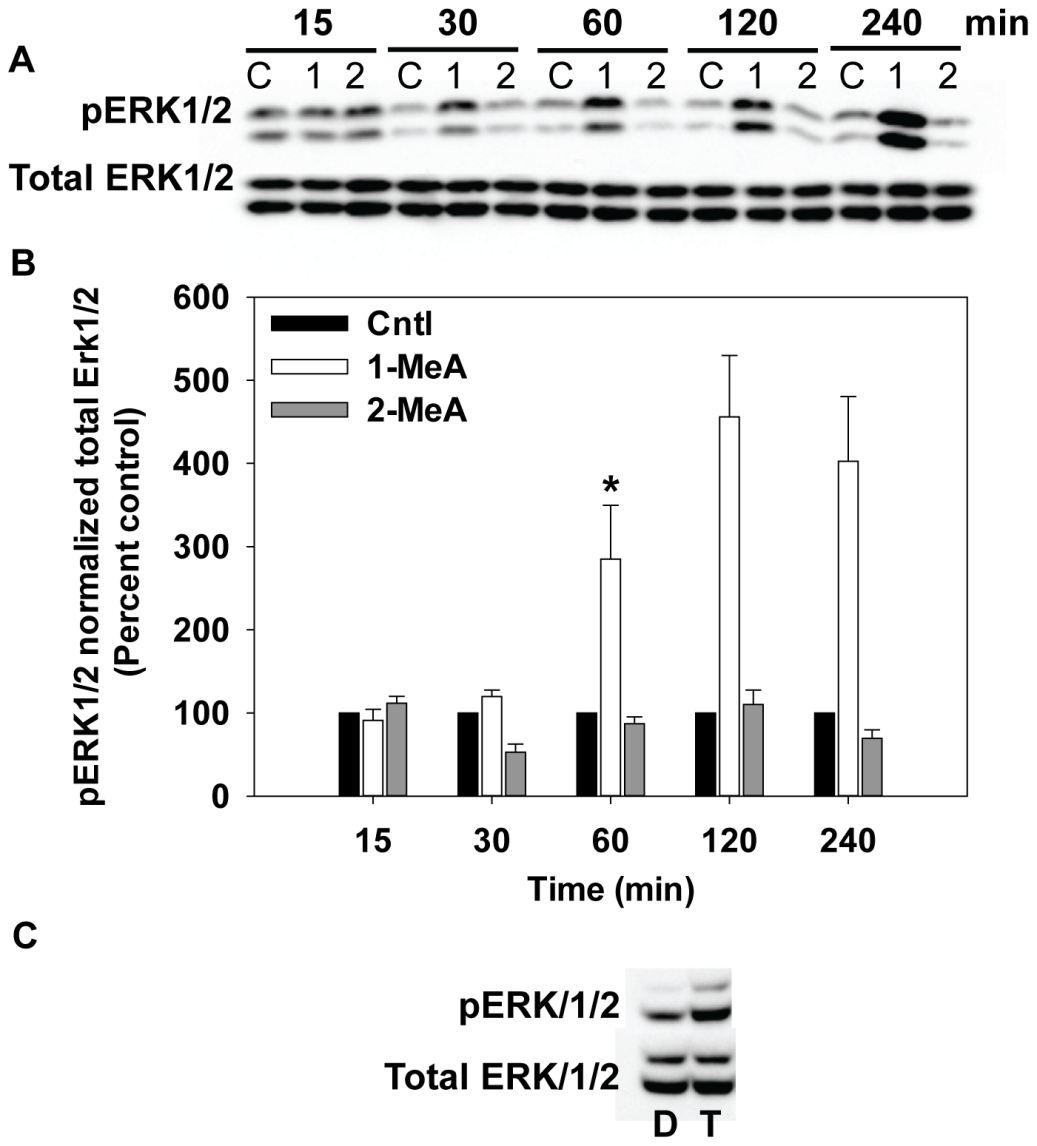
Phospho-ERK1/2 MAPK activation following treatment with 1- but not 2- MeA. (A) Immunoblot for pERK1/2 and total ERK for 15 min to 4 h of treatment. (B) Densitometric analysis of pERK1/2 normalized to total ERK, represented as percent control. Concentrations used: 75 µM 1-MeA and 75 µM 2-MeA. Mean ± SEM presented with n = 3 per study, replicated 3 times. 1-MeA, 1-methylanthracene; 2-MeA, 2-methylanthracene; Cntl, CH_3_CN vehicle control. ^*^p<0.05 compared to control; ^+^p<0.05 compared to 2-MeA treated cells at the same time point. (C) Positive control using T, TPA (50 nM, 60 min) or D, DMSO (negative control) for activation of ERK1/2.

**Figure 5:**
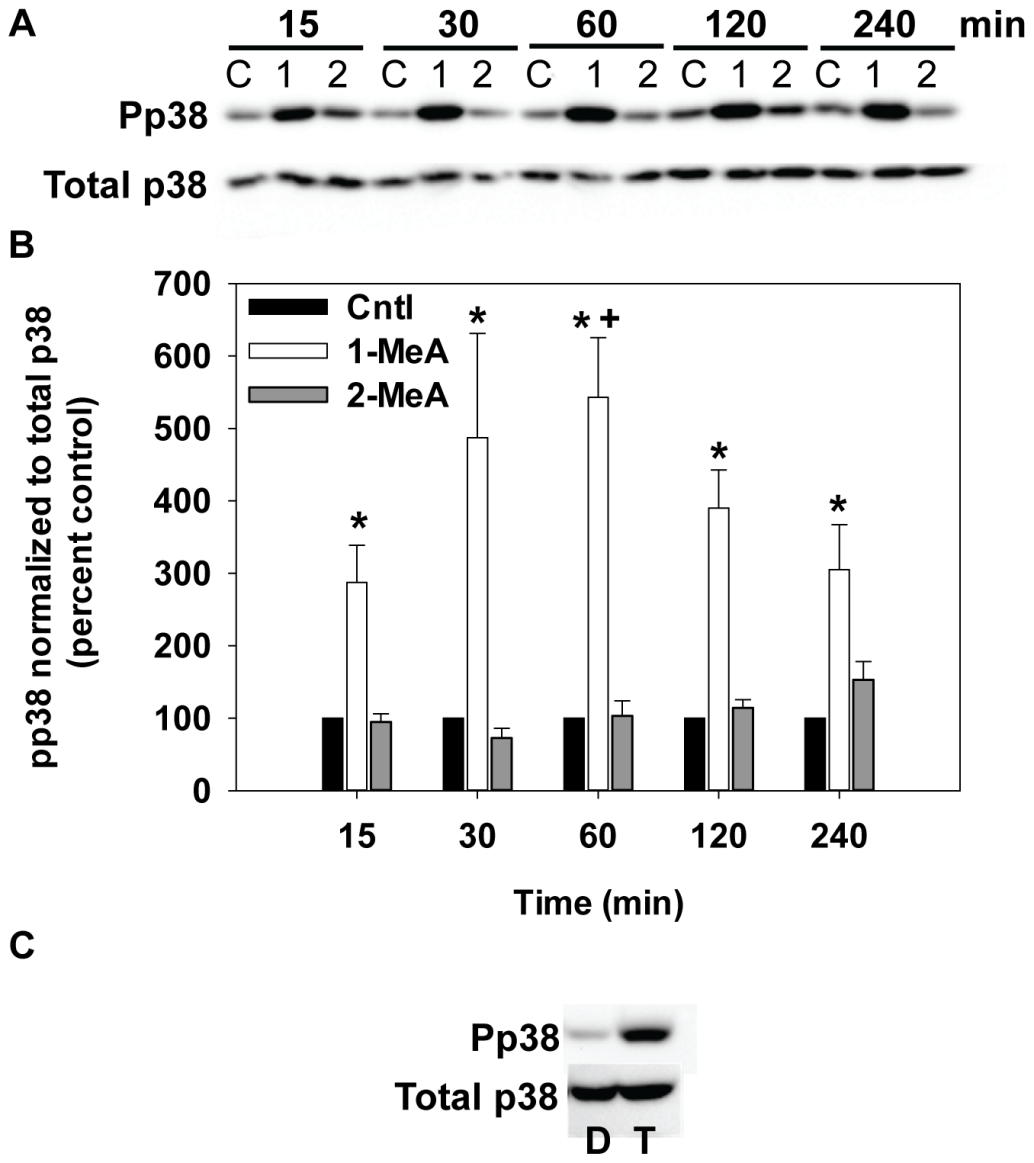
PhosphopP38 (pP38) MAPK activation following treatment with 1- but not 2- MeA. (A) Immunoblot for pP38 and total p38 for 15 min to 4 h of treatment. (B) Densitometric analysis of pP38 normalized to total P38, represented as percent control. Concentrations used: 75 µM 1-MeA and 75 µM 2-MeA. Mean ± SEM presented with n = 3 per study, replicated 3 times. 1-MeA, 1-methylanthracene; 2-MeA, 2-methylanthracene; Cntl, CH_3_CN vehicle control. ^*^p<0.05 compared to control; ^+^p<0.05 compared to 2-MeA treated cells at the same time point. (C) Positive control using T, TPA (50 nM, 60 min) or D, DMSO (negative control) for activation of P38.

The activity of MAP kinase was assessed over a time course of 15 min to 4 h using immunoblotting techniques of the phosphorylated (activated) forms of ERK and p38 (Fig. 4 and 5). Phosphorylated ERK1/2 (pERK1/2) was significantly increased at 1–4 h by 1-MeA while 2-MeA and the vehicle control (Cntl) had no effect on the activation of pERK1/2 (Fig. 4). Phosphorylated P38 (pP38) also rapidly increased following exposure to 1-MeA, but not to exposure with 2-MeA or the vehicle (Fig. 5). The phosphorylation of P38 occurred within 15 min of exposure to 1-MeA, peaked at 1 h, and persisted through 4 h. Significant differences were observed for both pERK1/2 and pP38 after normalization to total ERK and total P38 in response to 1-MeA for both treatment and time, but not to 2-MeA or control treatments (Fig. 4B and 5B). TPA was used as a positive control for pERK1/2 and pP38 in these studies, as observed in part C of Figures 4 and 5. Twenty-five and 50 µM doses of 1-MeA were also used to demonstrate increased activation of ERK1/2 at lower doses (Fig. S1).

### Cx43 Expression

**Figure 6:**
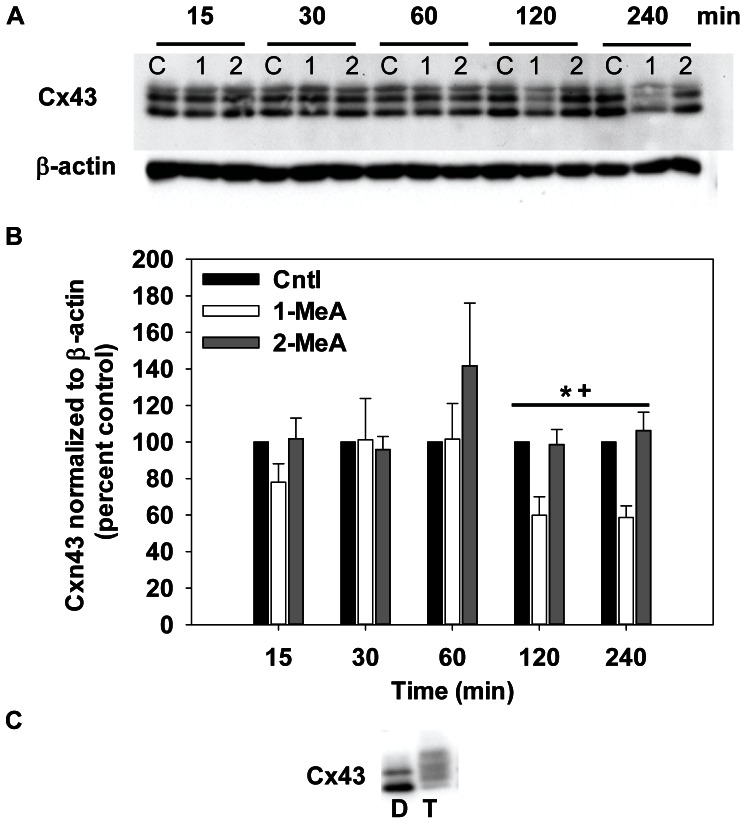
Decreases in Cx43 protein expression following 1-MeA treatment but not 2-MeA treatment. (A) Immunoblot of Cx43 for 15 min to 4 h after treatment. (B) Densitometric analysis of Cx43 normalized to β-actin, represented as percent control. Concentrations used: 75 µM 1-MeA and 75 µM 2-MeA. Mean ± SEM presented with n = 3 per study, replicated 3 times. 1-MeA, 1-methylanthracene; 2-MeA, 2-methylanthracene; Cntl, CH_3_CN vehicle control. ^*^p<0.05 compared to control; ^+^p<0.05 compared to 2-MeA treated cells at the same time point. (C) Positive control using T (TPA; 50 nM, 60 min) or D (DMSO; negative control) for activation of Cx43.

Immunoblot analyses were used to assess the relative protein levels and phosphorylation status of Cx43, the major gap junction protein of these pulmonary cells. The same time course was used for Cx43 as the MAPK (15 min–4 h). As observed with previous endpoints, CH_3_CN control had no effect on Cx43 protein expression levels, similar to the lack of effect on GJIC or MAPK activation (Fig. 6). There were no significant changes in the ratios of the three forms of Cx43 at 15 and 30 min, which indicate no change in phosphorylation of the connexins and are within the time period of 1-MeA-induced dysregulation of GJIC. However, 1-MeA did significantly reduce total Cx43 expression at the 2 and 4 h time points as compared to either the vehicle or 2-MeA exposure (Fig. 6). In contrast, TPA induced a hyperphosphorylation of the connexins as indicated by a mobility shift to higher molecular weights (Fig. 6C). This latter result indicated that Cx43 in lung cells are capable of phosphorylation changes, yet the 1-MeA had no effect on the phosphorylation status as determined by the lack of mobility shifts in immunoblots.

**Figure 7:**
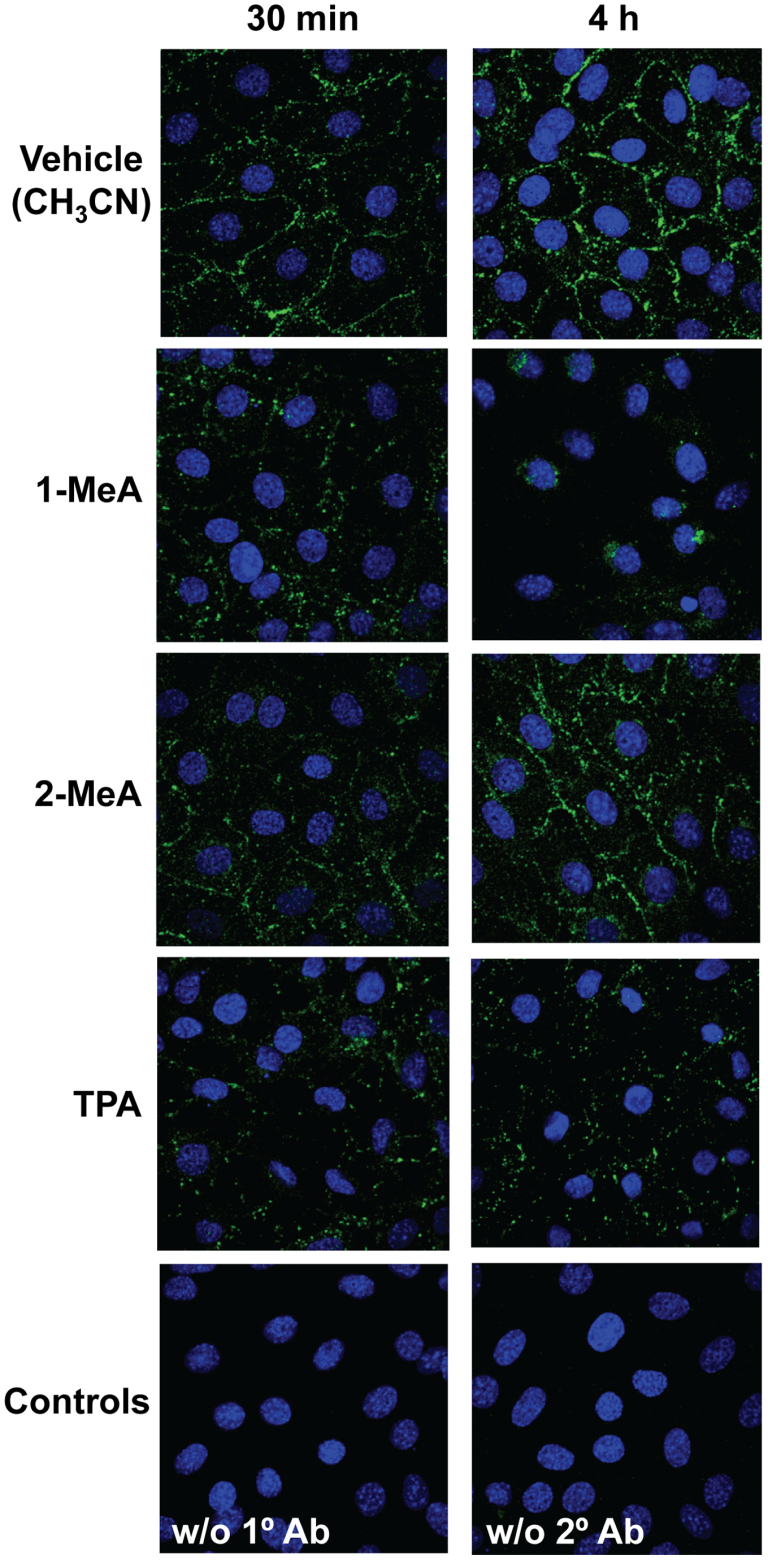
Reduced C10 cell Cx43 immunostaining in response to 1-MeA, but not 2-MeA treatment. Cells were treated with acetonitrile vehicle (CH_3_CN), 1-MeA (75 µM), 2-MeA (75 µM), TPA (50 nM, 60 min) as a positive control, and two negative controls, one without (w/o) primary antibody and one without (w/o) secondary antibody. Two time points were assessed, 30 min and 4 h to observe changes at several time points known to inhibit gap junctions. Magnification was at 1000X using a Nikon D-Eclipse C1 confocal microscope. Experiments were repeated twice.

Cx43 immunostaining demonstrated punctate staining on the cell membrane in the vehicle or 2-MeA treated C10 cells at both 30 min and 4 h time points, however, in the 1-MeA exposed cells, the punctate staining decreased in the plasma membranes, became more apparent in the cytoplasm after 30 min, and almost completely disappeared by 4 h (Fig. 7). These events occurred after the dysregulation of GJIC and are similar to that observed with TPA in which dysfunctional GJIC lead to the connexins being retrafficked to the cytosolic cell compartments (Fig. 7).

### Role of MAPK in the Dysregulation of GJIC

**Figure 8:**
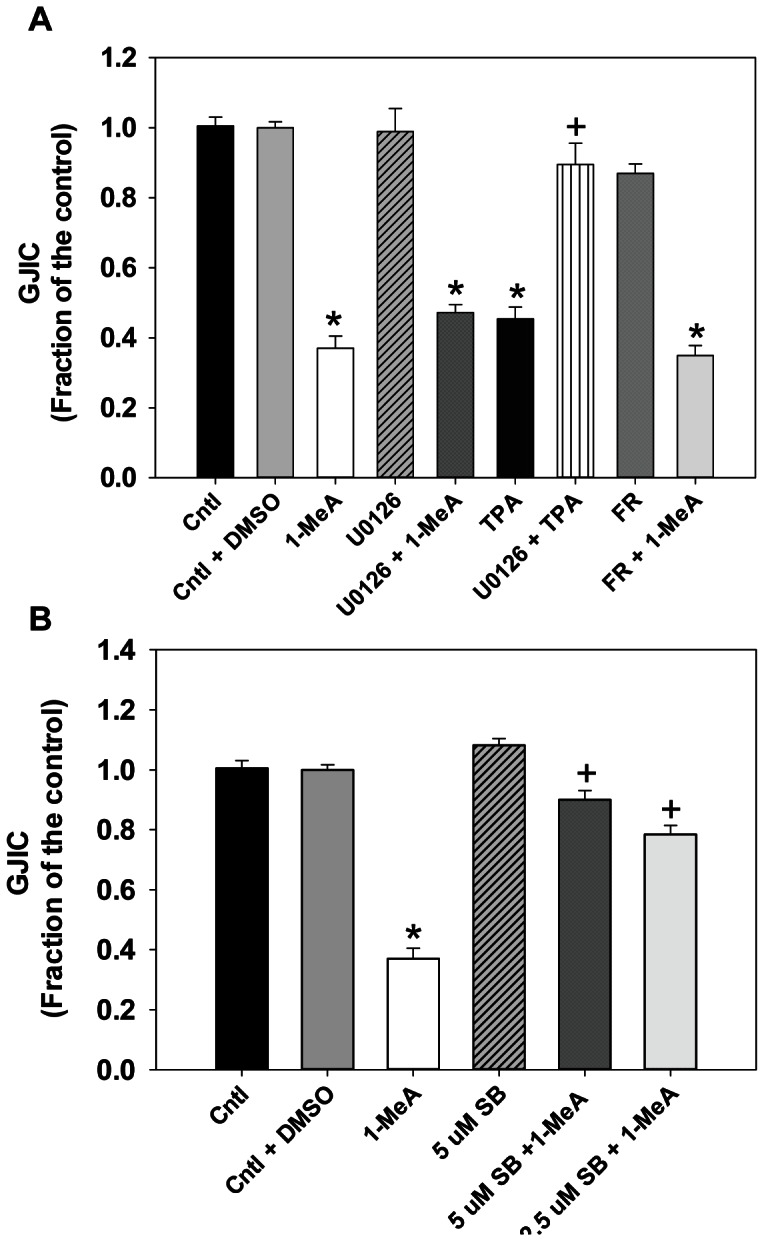
Reversal of 1-MeA-induced GJIC dysregulation using a p38 MAPK inhibitor. (A) U0126 (20 µM) and FR180204 (5 µM) were used to inhibit MEK or ERK1/2, respectively, prior to exposure to 1-MeA for 30 min. SL/DT assays were then done following 1-MeA treatment. TPA was also used as a positive control. Cntl, acetonitrile control; 1-MeA, 1-methylanthracene (75 µM, 30 min); U0126 alone (20 µM, 1 h prior); U0126+1-MeA, U0126 prior to incubation with 1-MeA; TPA (50 nM, 1 h), TPA alone; U0126+ TPA, U0126 prior to incubation with TPA; FR, FR180204 alone (5 µM, 1 h prior); FR +1-MeA, FR180204 prior to incubation with 1-MeA. Mean ± SEM presented with n = 3 per study, replicated 3 times. ^*^p<0.05 compared to control;^+^p<0.05 compared to TPA treated cells. (B) SB203580 (2.5, 5 µM) was used to inhibit p38 1 h prior to exposure to 1-MeA for 30 min. SL/DT assays were then done following 1-MeA treatment. SB, SB203580 (2.5 or 5 µM, 1 h); SB +1-MeA, SB203580 prior to incubation with 1-MeA. ^*^p<0.05 compared to control; ^+^p<0.05 compared to 1-MeA treated cells.

Because the activation of P38 and ERK1/2 in response to 1-MeA occurred at time points when GJIC was also inhibited, we determined the effects of ERK1/2 and P38 dysregulation on GJIC activity. We pre-incubated the cells with either a MEK-inhibitor (U0126; inhibits upstream of ERK1/2), a specific ERK1/2 inhibitor (FR180204; inhibits ERK1 and 2) or a specific inhibitor of p38 (SB203580) and then assessed the effects of 1-MeA on GJIC. Inhibition of P38 prevented the dysregulation of GJIC by 1-MeA, while inhibition of the MEK pathway using U0126 and ERK1/2 using FR180204, had no effect on the dysregulation of GJIC elicited by 1-MeA (Fig. 8). An additional P38 inhibitor (SB202190, 20 µM, [25]) was also used to reverse 1-MeA-induced dysregulation of GJIC and confirmed the SB203580 results (data not shown). Immunoblots of MAPKAPK-2, a known substrate for P38 [51] were done to demonstrate inhibition of P38, since SB203580 inhibits p38 activity, but not necessarily phosphorylation [47] (see Fig. S2A). pERK1/2 immunoblots also demonstrated inhibition of pERK with both U0126 and FR180204, although, FR180204 only partially inhibited ERK (Fig. S2B). These results indicate 1-MeA-induced dysregulation of GJIC depended, in part, on the p38, but not the ERK1/2 MAPK pathway. TPA-induced dysregulation of GJIC was prevented by U0126, the MEK inhibitor, which supports previous findings on the involvement of ERK1/2 MAPK in gap junction regulation with TPA [52].

### Inflammatory Pathways Downstream of MAPK

**Figure 9:**
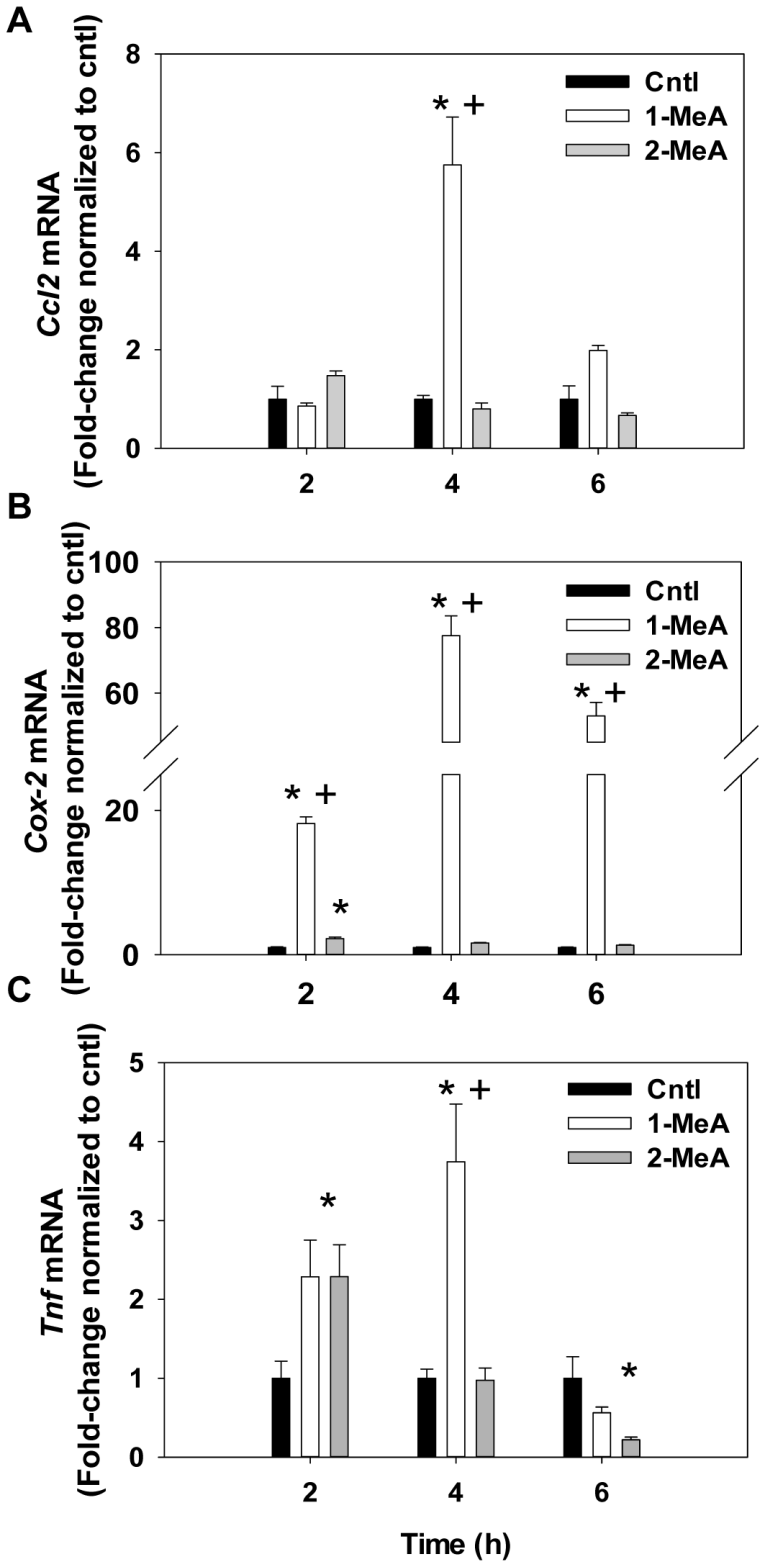
Inflammatory pathways downstream of MAPKs and dysregulation of GJIC are influenced by 1-MeA. (A) *Ccl2*, or *Mcp-1*, mRNA expression in response to 1-MeA, 2-MeA, or control treated cells. (B) *Ptgs2*, or *Cox2*, mRNA expression in response to 1-MeA, 2-MeA, or control treated cells. (C) *Tnf* mRNA expression in response to 1-MeA, 2-MeA, or control treated cells. Concentrations used: 1-MeA, (75 µM) and 2-MeA (75 µM). For each gene, mean and SEM are presented; n = 3 per treatment, repeated twice. Genes are first normalized to 18S followed by normalization to acetonitrile control per time point. Cntl = control acetonitrile; 1-MeA, 1-methylanthracene; 2-MeA, 2-methlyanthracene; Ccl2, chemokine (CC-motif) ligand 2; Mcp-1, monocyte chemoattractant 2; Cox-2, cyclooxygenase 1; Ptgs2, prostaglandin-endoperoxide synthase 2; Tnf, tumor necrosis factor α.

RNA expression for several pathways downstream of the MAPKs, among other pathways, was analyzed via qPCR for 2–6 h following 1- or 2-MeA treatments compared to vehicle treated cells at each time point (Fig. 9). *Ccl2* (*Mcp-1*; Fig. 9A) gene expression significantly increased at 4 h and was increased at 6 h following 1-MeA treatment, but was not observed in 2-MeA or vehicle treatments. *Cox2* (*Ptgs2;* Fig. 9B) expression was also highly elevated (between 18–80-fold; P<0.05) at 2, 4 and 6 h time points compared to either 2-MeA or control treatments. *Cox-2* expression was slightly elevated at 2 h in the 2-MeA treatment group. *Tnf* (Fig. 9 C) was also significantly elevated at 2 h in both 1- and 2-MeA compared to the vehicle treatment and remained significantly elevated only in the 1-MeA treatment at 4 h. However, by 6 h, repression of *Tnf* was observed in the 1- and 2-MeA treatment groups, suggesting feedback regulation of *Tnf* after the initial increase.

## Discussion

These novel studies address the effects of low molecular weight PAHs (1 and 2-MeA) on alveolar type II cells and identified several markers of downstream signaling pathways that were modulated in response to 1-MeA, but not 2-MeA. GJIC was dysregulated and MAPKs were activated (P38, pERK1/2) as an early response to the active PAH (1-MeA), but not to 2-MeA, suggesting a structure-activity relationship underlies the efficacy of responses. Longer-term effects included a decrease in the protein levels of Cx43, relocalization of Cx43 from the plasma membrane to the cytoplasm, and the induction of specific inflammatory mediators (*Mcp-1*, *Cox-2*, and *Tnf*). The rapid onset of GJIC dysregulation in our model at time points that coincide with observed increases in pP38 suggests an interaction between the MAPK pathways and regulation of gap junctions. Our finding that inhibition of P38 almost completely blocked the 1-MeA-induced dysregulation of gap junctions, indicates that the regulation of GJIC was, in fact, P38-dependent. In response to 1-MeA, the activation of pERK1/2 was subsequent (30 min) to the dysregulation of GJIC (<15 min), and the inhibition of MEK or ERK1/2 with U0126 or FR180204, respectively, did not prevent the dysregulation of GJIC, which indicates that the ERK1/2 pathway was not involved in 1-MeA-induced dysregulation of GJIC. Cx43 protein levels decreased several hours after the dysregulation of GJIC, although the Cx43 staining at 30 min (Fig. 7) indicated early removal of Cx43 from the plasma membrane of treated cells, consistent with Cx43 trafficking. However, we cannot rule out the possibility that other connexins are involved in this 1-MeA-induced response. Alveolar type II cells can also express Cx26, Cx32, Cx37, and Cx46 and some can heterodimerize, although the concept of mixed gap junction channels remains controversial [29].

### Gap Junctions and the Lung

Gap junctions have important roles in many normal physiological processes in the CNS, kidney, liver, heart, and lung, [29], [53], [54], and when channel resistance increases, impairment of function can lead to pathological states. In some lung diseases, such as acute lung injury, deficient communication between cells could be a causal mechanism. In lung fibroblasts isolated from idiopathic pulmonary fibrosis patients (IPF), gap junction activity and Cx43 expression was significantly decreased compared to controls [21], however the importance of decreased fibroblast communication in fibrosis is not clear.

Changes in the phosphorylation status of the connexin proteins have been implicated in the dysregulation of GJIC [55]–[58]. Our results indicate that 1-MeA did not alter the phosphorylation status of Cx43. TPA was used as the positive control for hyperphosphorylation of Cx43, as indicated in a shift of the bands to higher molecular weights and the disappearance of the bottom band (unphosphorylated Cx43) (Fig. 6C). The lack of an effect on the phosphorylation of Cx43 by 1-MeA persisted after the activation of ERK1/2 and p38, similar to findings for 1-MeA treated rat liver cells [25], [43] and p38 [25]. Although Cx43 has consensus sequences for MAPKs [59], [60], these results indicate that other signaling factors, which were not activated in response to 1-MeA, are also required to phosphorylate Cx43. The phosphorylation of Cx43 by TPA is known to be dependent on protein kinase C and ERK1/2 [61]. Alternatively, our results did show a decrease in all Cx43 bands that was consistent with our immunostaining experiments in which Cx43 localized to the cytoplasm, and displayed an overall decrease to near disappearance over our time course (Fig. 7).

### PAHs

The structural differences between 1- and 2-MeA while minor in appearance, elicit completely different responses in the C10 cells due to the lack of a bay-like region in the 2-MeA structure compared to the 1-MeA structure. Similar differences were observed in other cell types, such as liver and pancreas [25], [62], thus these structural differences are not unique to lung.

Doses for these studies were chosen based on several studies demonstrating levels of LMW PAHs in sidestream smoke (ETS) were>mainstream smoke (3186–5695 ng/cigarette) [63], [64]. At the low end of the range, 20 cigarettes/pack×3186 ng/cigarette×7d/wk = ∼ 0.45 mg/wk for the PAH mixture released as ETS. Our dose range is 0.03–0.04 mg/study per individual PAH, however, doses for future studies in mixtures will likely be lower, due to contributing effects [65]. These doses and PAHs were chosen to identify any differences in the phenotypes assessed between an active and inactive isomer, however, we are typically exposed to mixtures of multiple PAHs and not single PAHs. For example, coal tar pitch contains many PAHs [66], both LWM, such as methylanthracenes, and high MW PAHs, and recently was shown to induce promoting characteristics in lung cells [67]. Furthermore, a mixture of PAHs commonly found in coal tar and creosote products demonstrated that dysregulation of GJIC by these PAHs were additive [65], thus there is a definite cumulative effect by many of the PAHs indicating that doses should be based on total PAH content.

### MAPK Activation and Gap Junctions

TPA, a classic tumor promoter in most cell types, dysregulates gap junctions through several mechanisms, including MAPKs (P38 and ERK1/2) via the activation of PKC [61], [68], [69]. Thus, some MAPK pathways are known to mediate the regulation of GJIC in several cell types [61], [70]. ERK1/2 also dysregulates gap junctions in other classic non-genotoxic carcinogen models, such as perfluoroalkanoates [71]. However, the activation of ERK1/2 by 1-MeA was not involved in 1-MeA-induced dysregulation of GJIC in the lung cells used in this study, while P38 was involved. This suggests that ERK1/2 is independent of GJIC dysregulation, but is still activated by treatment with 1-MeA, while P38 is linked to GJIC shown by return of normal GJIC by inhibition of P38. Activation of P38 and ERK1/2 by 1-MeA is most likely through an indirect mechanism, eg. activation of receptors or G-proteins.

### Gap Junction and MAPK Involvement in Regulating the Pulmonary Microenvironment

In most lung diseases, the pulmonary microenvironment is critical in regulating the inflammation and adverse effects that occur, such as fibrosis and carcinogenesis. Inflammation is an important element of tumorigenesis, at all stages, and is typically considered downstream of earlier triggering events, such as dysregulation of gap junctions and activation of MAPKs [23], [72], [73].

P38 can induce Mcp-1 (Ccl2) in endothelial cells [74], [75], and induce other chemokines, such as IL-8, in lung epithelial cells [76]. Cx43 decreased Mcp-1 expression in glioblastoma cells which lead to decreased proliferation, suggesting that a reduction in gap junction function lead to increased Mcp-1 [77]. Ccl2 (Mcp-1) is routinely used as a marker in our *in vivo* promotion models, such as BHT and vanadium pentoxide (V_2_O_5_) [39], [50], because it is secreted by the pulmonary epithelial cells as a chemoattractant for both macrophages and lymphoctyes into the lung. Macrophage infiltration plays a pivotal role during promotion in these *in vivo* models and an *in vivo* pilot study using fluoranthene, another LMW PAH, demonstrated increased numbers of bronchoalveolar lavage macrophages in the lungs compared to controls (unpublished data, A.K. Bauer). V_2_O_5_-induced promotion also leads to increases in both ERK1/2 and P38 MAPK activation [39]. Thus, in our *in vitro* model, we show that *Mcp-1* is increased following MAPK activation (P38 and ERK1/2), and gap junction dysregulation, all evidence for 1-MeA’s potential to contribute to the early signaling events involved in promoting lung cancer.

P38 is also considered the major MAPK involved in TNF-induction [78], [79]. Interestingly, TNF can also induce MAPK [80], particularly P38, thus, a positive feed-forward mechanism may be involved in regulating this pathway. In addition, TNF can inhibit gap junctions in fibroblasts [81] and repress Cx43 expression in keratinocytes [82], thus supporting a possible link between pro-inflammatory cytokine expression and regulation of gap junctions.

In rat liver cells (WB F344) and human coronary artery endothelial cells, arachidonic acid (AA) is released in response to 1-MeA, but not 2-MeA [25], [83]. Since Cox-2 is downstream of AA and cytosolic phospholipase A_2_ (cPLA_2_) [23], we determined the possible involvement of the Cox-2 pathway in our model. Prostaglandin E2 (PGE_2_) and prostaglandin I_2_ (PGI_2_) production, both downstream of Cox-2, are ERK1/2-dependent in response to TNF and interferon (IFN)γ stimulation in C10 cells as well as nitric oxide production and inducible nitric oxide synthase (iNOS) expression [17], [19]. Preliminary data from our lab demonstrated that both p38 and ERK1/2 regulate Cox-2 gene expression in 1-MeA treated C10 cells (data not shown).

### Conclusion

Although the most abundant PAHs in the environment are the LMW PAHs, research on PAHs has focused primarily on the mutagenic and DNA damaging properties of the higher molecular weight compounds, however, evidence supports the LMW PAHs ability to induce molecular events relevant to lung injury, inflammation, and tumor promotion. We previously reported the effects of 41 different PAHs on GJIC in rat liver cell lines [41], [42], [65], [84] where most of these PAHs, including the high molecular weight PAHs, dysregulated GJIC with varying potencies [41], [42]. However, the potencies of the LMW PAHs (4 rings or less) tended to be much stronger if they contained a bay or bay-like region [11], [41], [42].

Our results extend this research into a lung derived cell model system in which the PAH containing a bay-like structure was biologically active and the PAH lacking these structures was inactive, consistent with the liver model cell systems. Considering that the lung is the major organ of exposure to PAHs in the environment, these novel results suggest that the LMW, non-genotoxic PAHs could contribute to the lung injury and promotion process, thus posing a potential human health risk as well as linking LMW PAH-induced effects with proinflammatory responses, which was also dependent on the PAH possessing a bay-like structure. The role of inflammation in all stages of cancer, fibrosis, COPD, and many other pulmonary diseases further demonstrates the importance of this link between PAH exposure, early signaling and inflammatory mediator involvement [85]–[88].

Collectively, this study provides the first assessment of the effects of these LMW PAHs on pulmonary cells that may lead to predictive outcomes for future identification of complex mixture effects, as well as identify novel targets for potential biomarkers, develop innovative chemopreventive strategies, and impact future risk assessment for these PAHs in lung and other cancers, such as liver, pancreas, and breast.

## Supporting Information

Figure S1 **pERK1/2 at several doses of 1-MeA demonstrating increased ERK1/2 activation.** C10 cells were exposed to 25 and 50 µM 1-MeA for 2 h and compared to DMSO vehicle control treated cells (0 µM). Densitometry of the immunoblot is presented with mean ± SEM. *P<0.05 for 1-MeA treated cells compared to DMSO alone.
(TIF)

Figure S2 **Confirmation of inhibition of p38 and ERK1/2 activity following inhibitor incubation using immunoblots.** (A) A MAPKAPK-2 immunoblot in C10 cells demonstrating inhibition of phosphorylation of MAPKAPK-2, a known substrate of p38, compare lane 3 to lane 5 and 6. Lane 1, control, acetonitrile; lane 2, control+DMSO; lane 3, 1-MeA (75 µM); lane 4, control+SB203580 inhibitor (5 µM); lane 5, SB203580 (5 µM) inhibitor +1-MeA; lane 6, SB203580 (2.5 µM) inhibitor +1-MeA. Total MAPKAPK-2 is seen below the phosphorylated immunoblot demonstrating equal amounts of total in each sample. (B) pERK1/2 immunoblot following treatment with FR180204 (5 µM) or U0126 (20 µM) prior to treatment with 75 µM 1-MeA demonstrating inhibition of ERK phosphorylation in lanes 5 and 6 compared to 3. Lane 1, control, acetonitrile; lane 2, control+DMSO; lane 3, 1-MeA (75 µM); lane 4, control+FR180204 (5 µM); lane 5 FR180204 (5 µM) +1-MeA; lane 6, U0126 (20 µM) +1-MeA.
(TIF)

## References

[1] Hazelton WD, Clements MS, Moolgavkar SH (2005) Multistage carcinogenesis and lung cancer mortality in three cohorts. Cancer Epidemiol Biomarkers Prev 14: 1171–1181. 15894668 10.1158/1055-9965.EPI-04-0756

[2] Klaunig JE, Kamendulis LM, Xu Y (2000) Epigenetic mechanisms of chemical carcinogenesis. Hum Exp Toxicol 19: 543–555. 11211991 10.1191/096032700701546442

[3] Trosko JE (2001) Commentary: is the concept of “tumor promotion” a useful paradigm? Mol Carcinog 30: 131–137. 11301473

[4] ATSDR (2005) Toxicology profile for polyaromatic hydrocarbons. Boca Raton, FL: CRC Press.

[5] Palitti F, Cozzi R, Fiore M, Palombo F, Polcaro C, et al (1986) An in vitro and in vivo study on mutagenic activity of fluoranthene: comparison between cytogenetic studies and HPLC analysis. Mutat Res 174: 125–130. 3713730 10.1016/0165-7992(86)90102-8

[6] Roszinsky-Kocher G, Basler A, Rohrborn G (1979) Mutagenicity of polycyclic hydrocarbons. V. Induction of sister-chromatid exchanges in vivo. Mutat Res 66: 65–67. 423906

[7] Wilson KM, Klein JD, Blumkin AK, Gottlieb M, Winickoff JP (2011) Tobacco-smoke exposure in children who live in multiunit housing. Pediatrics 127: 85–92. 21149434 10.1542/peds.2010-2046

[8] Centers for Disease C, Prevention (2012) Current tobacco use and secondhand smoke exposure among women of reproductive age - 14 countries, 2008–2010. MMWR Morb Mortal Wkly Rep 61: 877–882. 23114255

[9] He Y, Jiang B, Li LS, Li LS, Ko L, et al.. (2012) Secondhand smoke exposure predicted chronic obstructive pulmonary disease and other tobacco related mortality in a 17-years cohort study in China. Chest. 10.1378/chest.11-2884 22628493

[10] Miller RL, Garfinkel R, Horton M, Camann D, Perera FP, et al (2004) Polycyclic aromatic hydrocarbons, environmental tobacco smoke, and respiratory symptoms in an inner-city birth cohort. Chest 126: 1071–1078. 15486366 10.1378/chest.126.4.1071 PMC2223076

[11] Upham BL, Weis LM, Trosko JE (1998) Modulated gap junctional intercellular communication as a biomarker of PAH epigenetic toxicity: structure-function relationship. Environ Health Perspect 106 Suppl 4975–981. 9703481 10.1289/ehp.98106s4975 PMC1533337

[12] Warshawsky D, Barkley W, Bingham E (1993) Factors affecting carcinogenic potential of mixtures. Fundam Appl Toxicol 20: 376–382. 8504912 10.1006/faat.1993.1048

[13] Lloyd CM, Saglani S (2010) Asthma and allergy: the emerging epithelium. Nat Med 16: 273–274. 20208514 10.1038/nm0310-273 PMC3380503

[14] Fujino N, Ota C, Takahashi T, Suzuki T, Suzuki S, et al.. (2012) Gene expression profiles of alveolar type II cells of chronic obstructive pulmonary disease: a case-control study. BMJ Open 2. 10.1136/bmjopen-2012-001553 PMC3532994 23117565

[15] Schottenfeld D (2000) Etiology and Epidemiology of Lung Cancer. In: Pass HI, Mitchell JB, Johnson DH, Turrisi AT, Minna JD, editors. Lung Cancer- Principles and Practice. 2nd ed. Philadelphia: Lippincott Williams and Wilkins. 367–388.

[16] American Cancer Society (2012) What is non-small cell lung cancer? In: American Cancer Society Inc., editor. Available: http://www.cancer.org/cancer/lungcancer-non-smallcell/index. Accessed 16 December, 2012.

[17] Dwyer-Nield LD, Srebernak MC, Barrett BS, Ahn J, Cosper P, et al (2005) Cytokines differentially regulate the synthesis of prostanoid and nitric oxide mediators in tumorigenic versus non-tumorigenic mouse lung epithelial cell lines. Carcinogenesis 26: 1196–1206. 15746162 10.1093/carcin/bgi061

[18] Malkinson AM, Dwyer-Nield LD, Rice PL, Dinsdale D (1997) Mouse lung epithelial cell lines–tools for the study of differentiation and the neoplastic phenotype. Toxicology 123: 53–100. 9347924 10.1016/s0300-483x(97)00108-x

[19] Rice PL, Barrett BS, Fritz JM, Srebernak MC, Kisley LR, et al (2010) Regulation of cytokine-induced prostanoid and nitric oxide synthesis by extracellular signalregulated kinase 1/2 in lung epithelial cells. Exp Lung Res 36: 558–571. 20815659 10.3109/01902148.2010.491891 PMC3084151

[20] Thompson DC, Porter SE, Bauer AK, Das KC, Ou B, et al (1998) Cytokine-induced nitric oxide formation in normal but not in neoplastic murine lung epithelial cell lines. Am J Physiol 274: L922–932. 9609731 10.1152/ajplung.1998.274.6.L922

[21] Trovato-Salinaro A, Trovato-Salinaro E, Failla M, Mastruzzo C, Tomaselli V, et al (2006) Altered intercellular communication in lung fibroblast cultures from patients with idiopathic pulmonary fibrosis. Respir Res 7: 122. 17005044 10.1186/1465-9921-7-122 PMC1594576

[22] Bauer AK, Malkinson AM, Kleeberger SR (2004) Susceptibility to neoplastic and non-neoplastic pulmonary diseases in mice: genetic similarities. Am J Physiol Lung Cell Mol Physiol 287: L685–703. 15355860 10.1152/ajplung.00223.2003

[23] Bauer AK, Rondini EA (2009) Review paper: the role of inflammation in mouse pulmonary neoplasia. Vet Pathol 46: 369–390. 19176494 10.1354/vp.08-VP-0217-B-REV

[24] Rosenkranz M, Rosenkranz HS, Klopman G (1997) Intercellular communication, tumor promotion and non-genotoxic carcinogenesis: relationships based upon structural considerations. Mutat Res 381: 171–188. 9434874 10.1016/s0027-5107(97)00165-6

[25] Upham BL, Blaha L, Babica P, Park JS, Sovadinova I, et al (2008) Tumor promoting properties of a cigarette smoke prevalent polycyclic aromatic hydrocarbon as indicated by the inhibition of gap junctional intercellular communication via phosphatidylcholine-specific phospholipase C. Cancer Sci. 99: 696–705. 10.1111/j.1349-7006.2008.00752.x PMC3023995 18377422

[26] Upham BL, Weis LM, Rummel AM, Masten SJ, Trosko JE (1996) The effects of anthracene and methylated anthracenes on gap junctional intercellular communication in rat liver epithelial cells. Fundam Appl Toxicol 34: 260–264. 8954755 10.1006/faat.1996.0195

[27] Vang O, Wallin H, Autrup H (1995) Inhibition of intercellular communication by condensates of high and low tar cigarettes. Arch Toxicol 69: 415–420. 7495381 10.1007/s002040050193

[28] Guan X, Hardenbrook J, Fernstrom MJ, Chaudhuri R, Malkinson AM, et al (1995) Down-regulation by butylated hydroxytoluene of the number and function of gap junctions in epithelial cell lines derived from mouse lung and rat liver. Carcinogenesis 16: 2575–2582. 7586169 10.1093/carcin/16.10.2575

[29] Johnson LN, Koval M (2009) Cross-talk between pulmonary injury, oxidant stress, and gap junctional communication. Antioxid Redox Signal 11: 355–367. 18816185 10.1089/ars.2008.2183 PMC2933150

[30] Avanzo JL, Mesnil M, Hernandez-Blazquez FJ, Mackowiak, II, Mori CM, et al (2004) Increased susceptibility to urethane-induced lung tumors in mice with decreased expression of connexin43. Carcinogenesis 25: 1973–1982. 15166089 10.1093/carcin/bgh193

[31] Yan Z, Subbaramaiah K, Camilli T, Zhang F, Tanabe T, et al (2000) Benzo[a]pyrene induces the transcription of cyclooxygenase-2 in vascular smooth muscle cells. Evidence for the involvement of extracellular signal-regulated kinase and NF-kappaB. J Biol Chem 275: 4949–4955. 10671533 10.1074/jbc.275.7.4949

[32] Ji H, Wang Z, Perera SA, Li D, Liang MC, et al (2007) Mutations in BRAF and KRAS converge on activation of the mitogen-activated protein kinase pathway in lung cancer mouse models. Cancer Res 67: 4933–4939. 17510423 10.1158/0008-5472.CAN-06-4592

[33] Walters DM, Antao-Menezes A, Ingram JL, Rice AB, Nyska A, et al (2005) Susceptibility of signal transducer and activator of transcription-1-deficient mice to pulmonary fibrogenesis. Am J Pathol 167: 1221–1229. 16251407 10.1016/S0002-9440(10)61210-2 PMC1603773

[34] Moghaddam SJ, Ochoa CE, Sethi S, Dickey BF (2011) Nontypeable Haemophilus influenzae in chronic obstructive pulmonary disease and lung cancer. Int J Chron Obstruct Pulmon Dis 6: 113–123. 21407824 10.2147/COPD.S15417 PMC3048087

[35] Bernert H, Sekikawa K, Radcliffe RA, Iraqi F, You M, et al (2003) Tnfa and Il-10 deficiencies have contrasting effects on lung tumor susceptibility: gender-dependent modulation of IL-10 haploinsufficiency. Mol Carcinog 38: 117–123. 14587096 10.1002/mc.10151

[36] Duperron C (1997) Chemopreventive efficacies of aspirin and sulindac against lung tumorigenesis in A/J mice. Carcinogenesis 18: 1001–1006. 9163687 10.1093/carcin/18.5.1001

[37] Kisley LR, Barrett BS, Bauer AK, Dwyer-Nield LD, Barthel B, et al (2002) Genetic ablation of inducible nitric oxide synthase decreases mouse lung tumorigenesis. Cancer Res 62: 6850–6856. 12460898

[38] Meyer AM, Dwyer-Nield LD, Hurteau G, Keith RL, Ouyang Y, et al (2006) Attenuation of the pulmonary inflammatory response following butylated hydroxytoluene treatment of cytosolic phospholipase A2 null mice. Am J Physiol Lung Cell Mol Physiol 290: L1260–1266. 16443645 10.1152/ajplung.00182.2005

[39] Rondini EA, Walters DM, Bauer AK (2010) Vanadium pentoxide induces pulmonary inflammation and tumor promotion in a strain-dependent manner. Part Fibre Toxicol 7: 9. 20385015 10.1186/1743-8977-7-9 PMC2861012

[40] Wattenberg LW, Estensen RD (1997) Studies of chemopreventive effects of budenoside on benzo[a]pyrene-induced neoplasia of the lung of female A/J mice. Carcinogenesis 18: 2015–2017. 9364014 10.1093/carcin/18.10.2015

[41] Blaha L, Kapplova P, Vondracek J, Upham B, Machala M (2002) Inhibition of gap-junctional intercellular communication by environmentally occurring polycyclic aromatic hydrocarbons. Toxicol Sci 65: 43–51. 11752684 10.1093/toxsci/65.1.43

[42] Weis LM, Rummel AM, Masten SJ, Trosko JE, Upham BL (1998) Bay or baylike regions of polycyclic aromatic hydrocarbons were potent inhibitors of Gap junctional intercellular communication. Environ Health Perspect 106: 17–22. 9417772 10.1289/ehp.9810617 PMC1532939

[43] Rummel AM, Trosko JE, Wilson MR, Upham BL (1999) Polycyclic aromatic hydrocarbons with bay-like regions inhibited gap junctional intercellular communication and stimulated MAPK activity. Toxicol Sci 49: 232–240. 10416268 10.1093/toxsci/49.2.232

[44] Wan B, Sayler GS, Schultz TW (2006) Structure-activity relationships for flow cytometric data of smaller polycyclic aromatic hydrocarbons. SAR QSAR Environ Res 17: 597–605. 17162389 10.1080/10629360601033374

[45] Upham BL (2011) Role of integrative signaling through gap junctions in toxicology. Curr Protoc Toxicol Chapter 2: Unit2 18. 10.1002/0471140856.tx0218s47 PMC3074483 21400682

[46] Chaudhuri R, Sigler K, Dupont E, Trosko JE, Malkinson AM, et al (1993) Gap junctional intercellular communication in mouse lung epithelial cell lines: effects of cell transformation and tumor promoters. Cancer Lett 71: 11–18. 8395972 10.1016/0304-3835(93)90090-v

[47] Tong Q, Zheng L, Dodd-o J, Langer J, Wang D, et al (2006) Hypoxia-induced mitogenic factor modulates surfactant protein B and C expression in mouse lung. Am J Respir Cell Mol Biol 34: 28–38. 16166744 10.1165/rcmb.2005-0172OC PMC2644189

[48] Cho HY, Jedlicka AE, Reddy SP, Kensler TW, Yamamoto M, et al (2002) Role of NRF2 in protection against hyperoxic lung injury in mice. Am J Respir Cell Mol Biol 26: 175–182. 11804867 10.1165/ajrcmb.26.2.4501

[49] Bauer AK, Cho HY, Miller-Degraff L, Walker C, Helms K, et al (2011) Targeted deletion of Nrf2 reduces urethane-induced lung tumor development in mice. PLoS One 6: e26590. 22039513 10.1371/journal.pone.0026590 PMC3198791

[50] Bauer AK, Fostel J, Degraff LM, Rondini EA, Walker C, et al (2009) Transcriptomic analysis of pathways regulated by toll-like receptor 4 in a murine model of chronic pulmonary inflammation and carcinogenesis. Mol Cancer 8: 107. 19925653 10.1186/1476-4598-8-107 PMC2785769

[51] Ben-Levy R, Leighton IA, Doza YN, Attwood P, Morrice N, et al (1995) Identification of novel phosphorylation sites required for activation of MAPKAP kinase-2. EMBO J 14: 5920–5930. 8846784 10.1002/j.1460-2075.1995.tb00280.x PMC394711

[52] Ruch RJ, Trosko JE, Madhukar BV (2001) Inhibition of connexin43 gap junctional intercellular communication by TPA requires ERK activation. J Cell Biochem 83: 163–169. 11500965 10.1002/jcb.1227

[53] Eugenin EA, Basilio D, Saez JC, Orellana JA, Raine CS, et al (2012) The role of gap junction channels during physiologic and pathologic conditions of the human central nervous system. J Neuroimmune Pharmacol 7: 499–518. 22438035 10.1007/s11481-012-9352-5 PMC3638201

[54] Sorensen CM, Holstein-Rathlou NH (2012) Cell-cell communication in the kidney microcirculation. Microcirculation 19: 451–460. 22118633 10.1111/j.1549-8719.2011.00149.x

[55] Abdelmohsen K, Gerber PA, von Montfort C, Sies H, Klotz LO (2003) Epidermal growth factor receptor is a common mediator of quinone-induced signaling leading to phosphorylation of connexin-43: role of glutathione and tyrosine phosphatases. J Biol Chem 278: 38360–38367. 12874275 10.1074/jbc.M306785200

[56] Guan X, Wilson S, Schlender KK, Ruch RJ (1996) Gap-junction disassembly and connexin 43 dephosphorylation induced by 18 beta-glycyrrhetinic acid. Mol Carcinog 16: 157–164. 8688151 10.1002/(SICI)1098-2744(199607)16:3<157::AID-MC6>3.0.CO;2-E

[57] Husoy T, Cruciani V, Sanner T, Mikalsen SO (2001) Phosphorylation of connexin43 and inhibition of gap junctional communication in 12-O-tetradecanoylphorbol-13-acetate-exposed R6 fibroblasts: minor role of protein kinase C beta I and mu. Carcinogenesis 22: 221–231. 11181442 10.1093/carcin/22.2.221

[58] Musil LS, Goodenough DA (1991) Biochemical analysis of connexin43 intracellular transport, phosphorylation, and assembly into gap junctional plaques. J Cell Biol 115: 1357–1374. 1659577 10.1083/jcb.115.5.1357 PMC2289231

[59] Lampe PD, Lau AF (2004) The effects of connexin phosphorylation on gap junctional communication. Int J Biochem Cell Biol 36: 1171–1186. 15109565 10.1016/S1357-2725(03)00264-4 PMC2878204

[60] Warn-Cramer BJ, Lampe PD, Kurata WE, Kanemitsu MY, Loo LW, et al (1996) Characterization of the mitogen-activated protein kinase phosphorylation sites on the connexin-43 gap junction protein. J Biol Chem 271: 3779–3786. 8631994 10.1074/jbc.271.7.3779

[61] Rivedal E, Opsahl H (2001) Role of PKC and MAP kinase in EGF- and TPA-induced connexin43 phosphorylation and inhibition of gap junction intercellular communication in rat liver epithelial cells. Carcinogenesis 22: 1543–1550. 11532878 10.1093/carcin/22.9.1543

[62] Tai MH, Upham BL, Olson LK, Tsao MS, Reed DN Jr, et al (2007) Cigarette smoke components inhibited intercellular communication and differentiation in human pancreatic ductal epithelial cells. Int J Cancer 120: 1855–1862. 17266035 10.1002/ijc.22530

[63] Lee HL, Hsieh DP, Li LA (2010) Polycyclic aromatic hydrocarbons in cigarette sidestream smoke particulates from a Taiwanese brand and their carcinogenic relevance. Chemosphere 10.1016/j.chemosphere.2010.09.045. 10.1016/j.chemosphere.2010.09.045 20947129

[64] Moir D, Rickert WS, Levasseur G, Larose Y, Maertens R, et al (2008) A comparison of mainstream and sidestream marijuana and tobacco cigarette smoke produced under two machine smoking conditions. Chem Res Toxicol 21: 494–502. 18062674 10.1021/tx700275p

[65] Ghoshal B, Weber WJ, Rummel AM, Trosko JE, Upham BL (1999) Epigenetic Toxicity of a Mixture of Polycyclic Aromatic Hydrocarbonson Gap Junctional Intercellular Communication Before and After Biodegradation. Environment Science Technology 33: 1044–1050.

[66] Services USDoHaH (2002) Toxicological profile for wood, creosote, coal tar creosote, coal tar, coal tar pitch, and coal tar pitch volatiles. Atlanta, GA. 225–227 p.

[67] Feng F, Wu Y, Zhang S, Liu Y, Qin L, et al (2012) Macrophages Facilitate Coal Tar Pitch Extract-Induced Tumorigenic Transformation of Human Bronchial Epithelial Cells Mediated by NF-kappaB. PLoS One 7: e51690. 23227270 10.1371/journal.pone.0051690 PMC3515562

[68] Pahujaa M, Anikin M, Goldberg GS (2007) Phosphorylation of connexin43 induced by Src: regulation of gap junctional communication between transformed cells. Exp Cell Res 313: 4083–4090. 17956757 10.1016/j.yexcr.2007.09.010

[69] Woodruff ML, Chaban VV, Worley CM, Dirksen ER (1999) PKC role in mechanically induced Ca2+ waves and ATP-induced Ca2+ oscillations in airway epithelial cells. Am J Physiol 276: L669–678. 10198365 10.1152/ajplung.1999.276.4.L669

[70] Yang SR, Cho SD, Ahn NS, Jung JW, Park JS, et al (2005) Role of gap junctional intercellular communication (GJIC) through p38 and ERK1/2 pathway in the differentiation of rat neuronal stem cells. J Vet Med Sci 67: 291–294. 15805733 10.1292/jvms.67.291

[71] Upham BL, Park JS, Babica P, Sovadinova I, Rummel AM, et al (2009) Structure-activity-dependent regulation of cell communication by perfluorinated fatty acids using in vivo and in vitro model systems. Environ Health Perspect 117: 545–551. 19440492 10.1289/ehp.11728 PMC2679597

[72] Cohen BH, Diamond EL, Graves CG, Kreiss P, Levy DA, et al (1977) A common familial component in lung cancer and chronic obstructive pulmonary disease. Lancet 2: 523–526. 95731 10.1016/s0140-6736(77)90663-8

[73] Wu AH, Fontham ET, Reynolds P, Greenberg RS, Buffler P, et al (1995) Previous lung disease and risk of lung cancer among lifetime nonsmoking women in the United States. Am J Epidemiol 141: 1023–1032. 7771438 10.1093/oxfordjournals.aje.a117366

[74] Marin V, Farnarier C, Gres S, Kaplanski S, Su MS, et al (2001) The p38 mitogen-activated protein kinase pathway plays a critical role in thrombin-induced endothelial chemokine production and leukocyte recruitment. Blood 98: 667–673. 11468165 10.1182/blood.v98.3.667

[75] Sung FL, Slow YL, Wang G, Lynn EG, O K (2001) Homocysteine stimulates the expression of monocyte chemoattractant protein-1 in endothelial cells leading to enhanced monocyte chemotaxis. Mol Cell Biochem 216: 121–128. 11216856 10.1023/a:1017383916068

[76] Schmeck B, Zahlten J, Moog K, van Laak V, Huber S, et al (2004) Streptococcus pneumoniae-induced p38 MAPK-dependent phosphorylation of RelA at the interleukin-8 promotor. J Biol Chem 279: 53241–53247. 15485852 10.1074/jbc.M313702200

[77] Huang R, Lin Y, Wang CC, Gano J, Lin B, et al (2002) Connexin 43 suppresses human glioblastoma cell growth by down-regulation of monocyte chemotactic protein 1, as discovered using protein array technology. Cancer Res 62: 2806–2812. 12019157

[78] Brook M, Sully G, Clark AR, Saklatvala J (2000) Regulation of tumour necrosis factor alpha mRNA stability by the mitogen-activated protein kinase p38 signalling cascade. FEBS Lett 483: 57–61. 11033356 10.1016/s0014-5793(00)02084-6

[79] Lee JC, Laydon JT, McDonnell PC, Gallagher TF, Kumar S, et al (1994) A protein kinase involved in the regulation of inflammatory cytokine biosynthesis. Nature 372: 739–746. 7997261 10.1038/372739a0

[80] Kim EK, Choi EJ (2010) Pathological roles of MAPK signaling pathways in human diseases. Biochim Biophys Acta 1802: 396–405. 20079433 10.1016/j.bbadis.2009.12.009

[81] Ito S, Hyodo T, Hasegawa H, Yuan H, Hamaguchi M, et al (2010) PI3K/Akt signaling is involved in the disruption of gap junctional communication caused by v-Src and TNF-alpha. Biochem Biophys Res Commun 400: 230–235. 20727856 10.1016/j.bbrc.2010.08.045

[82] Tacheau C, Laboureau J, Mauviel A, Verrecchia F (2008) TNF-alpha represses connexin43 expression in HaCat keratinocytes via activation of JNK signaling. J Cell Physiol 216: 438–444. 18297686 10.1002/jcp.21412

[83] Tithof PK, Elgayyar M, Cho Y, Guan W, Fisher AB, et al (2002) Polycyclic aromatic hydrocarbons present in cigarette smoke cause endothelial cell apoptosis by a phospholipase A2-dependent mechanism. Faseb J 16: 1463–1464. 12205049 10.1096/fj.02-0092fje

[84] Upham BL, Masten SJ, Lockwood BR, Trosko JE (1994) Nongenotoxic effects of polycyclic aromatic hydrocarbons and their oxygenation by-products on the intercellular communication of rat liver epithelial cells. Fundam Appl Toxicol 23: 470–475. 7835547 10.1006/faat.1994.1129

[85] Allavena P, Mantovani A (2012) Immunology in the clinic review series; focus on cancer: tumour-associated macrophages: undisputed stars of the inflammatory tumour microenvironment. Clin Exp Immunol 167: 195–205. 22235995 10.1111/j.1365-2249.2011.04515.x PMC3278685

[86] Guadagni F, Ferroni P, Palmirotta R, Portarena I, Formica V, et al (2007) Review. TNF/VEGF cross-talk in chronic inflammation-related cancer initiation and progression: an early target in anticancer therapeutic strategy. In Vivo 21: 147–161. 17436563

[87] Smith LA, Paszkiewicz GM, Hutson AD, Pauly JL (2010) Inflammatory response of lung macrophages and epithelial cells to tobacco smoke: a literature review of ex vivo investigations. Immunol Res 46: 94–126. 20094822 10.1007/s12026-009-8133-6

[88] Van Laere S, Limame R, Van Marck EA, Vermeulen PB, Dirix LY (2010) Is there a role for mammary stem cells in inflammatory breast carcinoma?: a review of evidence from cell line, animal model, and human tissue sample experiments. Cancer 116: 2794–2805. 20503411 10.1002/cncr.25180

