# Supplementary figures and images for: Polycyclic Aromatic Hydrocarbon-Induced Signaling Events Relevant to Inflammation and Tumorigenesis in Lung Cells Are Dependent on Molecular Structure
Source: PLoS One. 2013 Jun 3;8(6):e65150. doi: 10.1371/journal.pone.0065150 (PMC3670909; doi:10.1371/journal.pone.0065150)

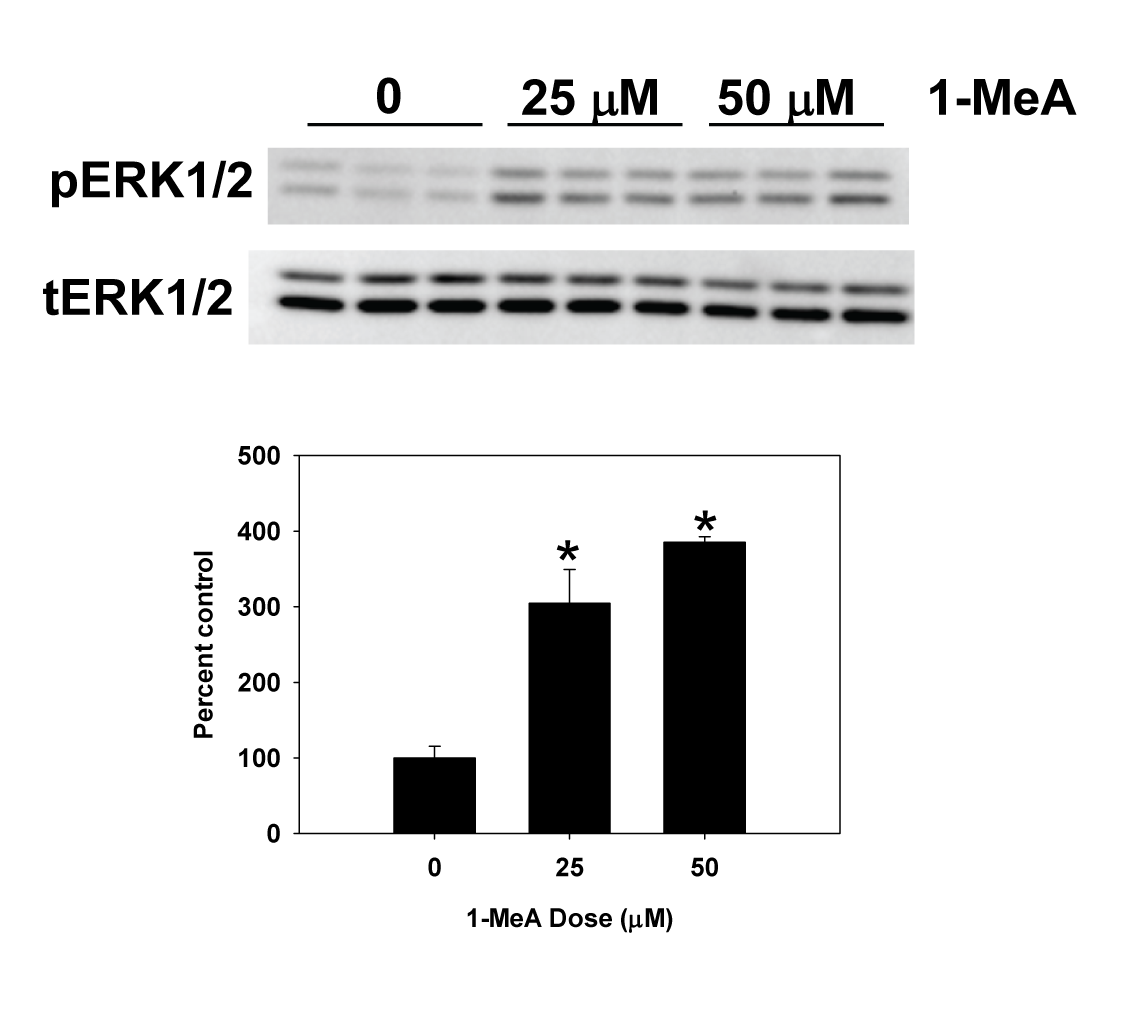

Supplement: Figure S1 — pERK1/2 at several doses of 1-MeA demonstrating increased ERK1/2 activation. C10 cells were exposed to 25 and 50 µM 1-MeA for 2 h and compared to DMSO vehicle control treated cells (0 µM). Densitometry of the immunoblot is presented with mean ± SEM. *P<0.05 for 1-MeA treated cells compared to DMSO alone. (TIF) [file pone.0065150.s001.tif]

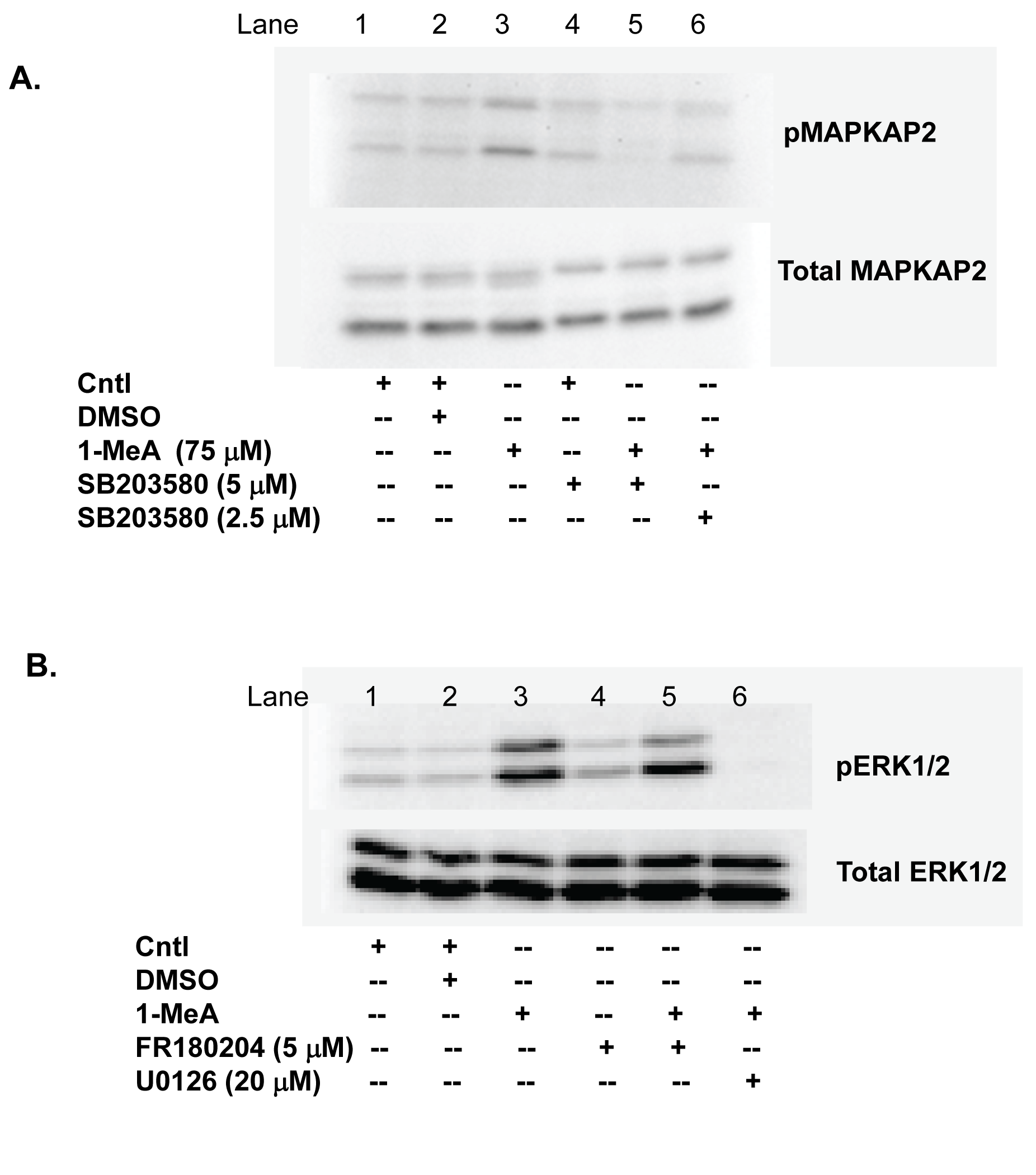

Supplement: Figure S2 — Confirmation of inhibition of p38 and ERK1/2 activity following inhibitor incubation using immunoblots. (A) A MAPKAPK-2 immunoblot in C10 cells demonstrating inhibition of phosphorylation of MAPKAPK-2, a known substrate of p38, compare lane 3 to lane 5 and 6. Lane 1, control, acetonitrile; lane 2, control+DMSO; lane 3, 1-MeA (75 µM); lane 4, control+SB203580 inhibitor (5 µM); lane 5, SB203580 (5 µM) inhibitor +1-MeA; lane 6, SB203580 (2.5 µM) inhibitor +1-MeA. Total MAPKAPK-2 is seen below the phosphorylated immunoblot demonstrating equal amounts of total in each sample. (B) pERK1/2 immunoblot following treatment with FR180204 (5 µM) or U0126 (20 µM) prior to treatment with 75 µM 1-MeA demonstrating inhibition of ERK phosphorylation in lanes 5 and 6 compared to 3. Lane 1, control, acetonitrile; lane 2, control+DMSO; lane 3, 1-MeA (75 µM); lane 4, control+FR180204 (5 µM); lane 5 FR180204 (5 µM) +1-MeA; lane 6, U0126 (20 µM) +1-MeA. (TIF) [file pone.0065150.s002.tif]
